# Evidence of polygenic regulation of the physiological presence of neurofilament light chain in human serum

**DOI:** 10.3389/fneur.2023.1145737

**Published:** 2023-03-08

**Authors:** Marisol Herrera-Rivero, Edith Hofer, Aleksandra Maceski, David Leppert, Pascal Benkert, Jens Kuhle, Reinhold Schmidt, Michael Khalil, Heinz Wiendl, Monika Stoll, Klaus Berger

**Affiliations:** ^1^Department of Genetic Epidemiology, Institute of Human Genetics, University of Münster, Münster, Germany; ^2^Department of Neurology, Medical University of Graz, Graz, Austria; ^3^Institute for Medical Informatics, Statistics and Documentation, Medical University of Graz, Graz, Austria; ^4^Neurologic Clinic and Polyclinic, MS Center and Research Center for Clinical Neuroimmunology and Neuroscience Basel (RC2NB), University Hospital of Basel, Basel, Switzerland; ^5^Clinical Trial Unit, Department of Clinical Research, University Hospital of Basel, Basel, Switzerland; ^6^Department of Neurology with Institute of Translational Neurology, University Hospital Münster, Münster, Germany; ^7^Department of Biochemistry, Genetic Epidemiology and Statistical Genetics, Maastricht University, Maastricht, Netherlands; ^8^Institute of Epidemiology and Social Medicine, University of Münster, Münster, Germany

**Keywords:** GWAS, neurofilament light chain, serum biomarkers, neuropathology, genetics

## Abstract

**Introduction:**

The measurement of neurofilament light chain (NfL) in blood is a promising biomarker of neurological injury and disease. We investigated the genetic factors that underlie serum NfL levels (sNfL) of individuals without neurological conditions.

**Methods:**

We performed a discovery genome-wide association study (GWAS) of sNfL in participants of the German BiDirect Study (*N* = 1,899). A secondary GWAS for meta-analysis was performed in a small Austrian cohort (*N* = 287). Results from the meta-analysis were investigated in relation with several clinical variables in BiDirect.

**Results:**

Our discovery GWAS identified 12 genomic loci at the suggestive threshold ((*p* < 1 × 10^−5^). After meta-analysis, 7 loci were suggestive of an association with sNfL. Genotype-specific differences in sNfL were observed for the lead variants of meta-analysis loci (rs34523114, rs114956339, rs529938, rs73198093, rs34372929, rs10982883, and rs1842909) in BiDirect participants. We identified potential associations in meta-analysis loci with markers of inflammation and renal function. At least 6 protein-coding genes (*ACTG2, TPRKB, DMXL1, COL23A1, NAT1*, and *RIMS2*) were suggested as genetic factors contributing to baseline sNfL levels.

**Discussion:**

Our findings suggest that polygenic regulation of neuronal processes, inflammation, metabolism and clearance modulate the variability of NfL in the circulation. These could aid in the interpretation of sNfL measurements in a personalized manner.

## Background

Neurofilament light chain (NfL) is a subunit of neurofilaments (NFs), cytoskeletal components found exclusively in neurons and particularly abundant in axons. NfL is a major component of the backbone of NFs in the central and peripheral nervous systems (1). Axonal damage and neuronal death due to neurological diseases, including those of inflammatory, neurodegenerative, traumatic and cerebrovascular nature, result in NfL release into the cerebrospinal fluid (CSF) and blood. Recent technological advances in immunoassay detection have enabled the accurate measurement of the small amounts of NfL that reach the circulation, facilitating its application as a universal peripheral biomarker of the presence and progression of neurological conditions, and of treatment responses (1–3). Therefore, investigating the factors that influence concentrations of NfL in the periphery becomes crucial for the interpretation of results. To date, it has been demonstrated that NfL serum levels (sNfL) increase with age (4) and potential confounding factors, such as body mass index and cardiovascular risk factors, have been suggested (5, 6).

Studies in population-based cohorts have shown a polygenic nature of numerous health-related serum biomarkers, including alanine transaminase (liver function), fibrinogen (clot formation) and glycated hemoglobin (type 2 diabetes mellitus), among many others. These findings can provide novel biological insights and facilitate disease diagnosis and stratification (7). Nevertheless, to our knowledge, no genetic associations with sNfL have been investigated. We hypothesized that the identification of genetic factors that modulate sNfL in physiological conditions will help interpretation on an individual basis, consequently improving the clinical applications of sNfL as a biomarker. To test our hypothesis, we performed a genome-wide association study (GWAS) and meta-analysis of sNfL in a total of 2,186 individuals of European descent without known neurological conditions, and correlated our findings with clinical data to identify potential sources of sNfL variability.

## Subjects and methods

### Study populations

The BiDirect Study was initiated in 2009 as a prospective, observational study integrating three cohorts: (1) community-dwelling adults (control cohort), (2) patients with an acute depressive episode (depression cohort), and (3) patients who recently suffered from acute myocardial infarction (MI cohort). The study, whose principal goal is the exploration of the bidirectional relationship between depression and subclinical arteriosclerosis, recruited participants in the district of Münster, Germany, and carried out extensive phenotyping and follow-up of all cohorts in parallel. The study design and methods have been previously described in detail (8). Here, we included 1,899 BiDirect participants (977 males, 922 females; mean age: 52.1 ± 7.9) from the control (763), depression (851) and MI (285) cohorts.

The Austrian Stroke Prevention Family Study (ASPS-Fam) cohort represents an extension of the prospective, population-based ASPS (Austrian Stroke Prevention Study) on the effects of vascular risk factors in normal aging. ASPS was established in 1991 in the city of Graz, Austria (9). For ASPS-Fam, first-degree relatives of ASPS participants were invited to join the study. The study's composition and inclusion criteria have been described elsewhere (10, 11). Here, we included 287 ASPS-Fam participants (115 males, 172 females; mean age: 64.3 ± 10.6).

The basic descriptive information of the BiDirect and ASPS-Fam cohorts are shown in Table 1. Summary information on study design and composition can be found in the Supplementary material 1. All participants of the BiDirect and ASPS-Fam cohorts provided written informed consent. Methods were carried out in accordance with the ethical standards laid down in the updated version of the 1964 Declaration of Helsinki. The BiDirect Study was approved by the Ethics Committee of the University of Münster and the Westphalian Chamber of Physicians in Münster, North-Rhine-Westphalia, Germany. The ASPS-Fam protocol was approved by the Ethics Committee of the Medical University of Graz, Austria.

**Table 1:** Basic description of BiDirect and ASPS-Fam cohorts.

**Cohort**	**Log2 sNfL (mean ±SD)**	**Age (mean ±SD)**	**Males (n)**	**Females (n)**	**Total (N)**
BiDirect	2.16 ± 0.45	52.1 ± 7.9	977	922	1,899
BiDirect-control	2.15 ± 0.44	53.4 ± 8.2	385	378	763
BiDirect-depression	2.13 ± 0.43	49.9 ± 7.3	348	503	851
BiDirect-MI	2.29 ± 0.5	55.2 ± 6.7	244	41	285
ASPS-Fam	4.99 ± 0.65	64.3 ± 10.6	115	172	287

MI, myorcardial infarction; sNfL, serum neurofilament light chain; SD, standard deviation.

### Serum measurements of NfL

Quantification of sNfL in BiDirect and ASPS-Fam was conducted at the University Hospital Basel, Switzerland, using the single molecule array (Simoa^®^) HDX analyzer (Quanterix, Lexington, MA, USA). In BiDirect participants, measurements of sNfL were obtained from non-fasting blood samples collected at the first visit, using the Simoa^®^ NF-light Advantage Kit. In ASPS-Fam participants, sNfL measurement (Supplementary material 1) has been previously described in detail (4). The sNfL values obtained at initial assessment were log2-transformed and used for all analyses herein reported. Therefore, with sNfL in our findings, we actually refer to log2 sNfL.

Because it is known that sNfL concentrations increase during aging (4), we tested for age-adjusted sex- and cohort-dependent sNfL differences in BiDirect using analysis of covariance (ANCOVA). We also tested for sNfL correlations, using the Pearson's method, with markers of inflammation, renal and liver function, lipids, hormones and brain volumes derived from magnetic resonance imaging (MRI) data (106 clinical variables in total). All *p* < 0.05 values were considered statistically significant. Here, age represented the age at participant recruitment, when baseline phenotyping (s0) took place. Clinical variables coming from up to three subsequent follow-up visits were identified as time points s2, s4, and s6.

### Genotype data

For BiDirect genotypes, genomic DNA was isolated from whole blood samples with EDTA using standard DNA extraction kits and procedures at the University of Münster. Genome-wide genotyping was performed with the Infinium PsychArray BeadChip v1 (Illumina) at Life&Brain GmbH (Bonn, Germany). Basic quality control (QC) was employed to remove samples and variants with high rates of missing data. This included removal of individuals with genotyping rate < 2%, cryptic relatedness (PI-HAT ≥1/16), sex mismatch and genetic outliers (distance in first two multidimensional scaling components >5 standard deviations from the mean), as well as the removal of variants with call rate < 2% and minor allele frequency (MAF) < 1%. Genotype imputation was performed with SHAPEIT (pre-phasing) (12) and IMPUTE2 (13) using the 1,000 Genomes Project, phase 3, European population reference panel (from here on, 1KG Reference Panel). Imputed variants were filtered for the INFO metric (≥0.8), MAF≥0.01 and Hardy-Weinberg equilibrium (HWE *p* ≥ 1 × 10^−6^). Individuals were further removed from the sample based on missing phenotypic data (age and baseline sNfL measurement). The final BiDirect GWAS dataset consisted of 5,597,244 genetic variants and 1,899 individuals.

For ASPS-Fam genotypes, genome-wide genotyping was performed with the Genome-Wide Human SNP Array 6.0 (Affymetrix). During the initial QC, variants with MAF < 0.05, HWE < 5 × 10^−6^ and low variant call rate (>2%) were excluded. Individuals with sex mismatch, cryptic relatedness, low sample call rate (>2%), a heterozygosity rate exceeding the mean ± 3 standard deviations and erroneous duplicates were removed. No genetic outliers were present. Genotype imputation was performed using the Michigan Imputation Server (14) and the 1KG Reference Panel.

Of note, genetic variants herein comprise single nucleotide polymorphisms (SNPs), as well as small insertions/deletions (indels) present in the datasets.

### Screening for genetic associations with sNfL

We conducted a discovery GWAS in the BiDirect dataset under an additive regression model, adjusting for age, sex, cohort and the first 10 principal components. A secondary GWAS in the smaller ASPS-Fam dataset was performed independently at the Medical University of Graz and was adjusted for age, sex and the first 10 principal components. After harmonization of summary statistics from both studies, we performed a weighted meta-analysis of all overlapping variants with Rsq≥0.8 and MAF≥0.01 using Plink 1.9 (15). Variants with high heterogeneity between studies (I>40 and Q < 0.1) were subsequently neglected.

### Definition of genomic loci for sNfL

For the discovery GWAS and the meta-analysis, we carried out downstream analyses on the FUMA GWAS platform (16) and defined genomic loci at the suggestive threshold of significance for genome-wide studies (*p* < 1 × 10^−5^), obtained variant annotations and identified the level of support for each signal. Linkage disequilibrium (LD) was defined by *r*^2^ ≥ 0.6 and a window of 500 kb, according to the 1KG Reference Panel. Subsequently, LD blocks were formed with variants under the suggestive threshold as lead variants, and containing all variants with *p* < 0.05 in the dataset that were in LD with the corresponding lead variants. Positional (gene) mapping was performed according to a maximum distance of 1 kb for the categories protein-coding, long non-coding RNA (lncRNA), non-coding RNA (ncRNA) and processed transcripts. Expression quantitative trait loci (eQTLs) were mapped using the BRAINEAC and GTEx v8 Brain databases. Only SNP-gene pairs with false discovery rate (FDR) < 0.05 were annotated.

### Functional implications of suggested candidate genes

To inform the biological meaning of our findings, we created a protein-protein interaction (PPI) network using our suggested meta-analysis candidate genes as input. The network was generated with the Gene Set analysis tool of the ReactomeFIViz app for Cytoscape v.3.7.1 (17, 18). Linker proteins and functional interaction (FI) annotations were incorporated into the network (version 2018). In addition, we performed clustering of nodes, as well as enrichment analyses of pathways and gene ontology cellular components (GO_CC) for each network cluster. Gene sets with FDR < 0.05 were considered significantly enriched.

### SNP heritability (h${}_{\text{SNP}}^{2}$)

We calculated the proportion of variance in sNfL concentrations explained by our discovery GWAS in BiDirect using the GREML-LDMS (LD- and MAF-stratified GREML) method implemented in GCTA (19, 20). For all autosomal variants with MAF≥0.01 in the imputed dataset, we calculated the 200 kb segment-based LD scores, stratified variants according to LD scores of individual SNPs, computed one genetic relationship matrix for each quartile of the stratified variants, and performed a restricted maximum likelihood analysis using these four matrices. The variance explained was adjusted for the same covariates as the GWAS. SNP heritability from our meta-analysis summary statistics was calculated using LDSC software (21) with LD scores pre-computed in 1KG Reference Panel data, as suggested by the authors.

### Screening for associations with clinical variables

For the lead variant of each loci resulting from our meta-analysis, we performed genotype-specific comparisons in BiDirect participants using an ANCOVA model adjusted for age. Moreover, for all variants within meta-analysis loci, we tested for associations with the same set of clinical variables used in the correlation analyses. These association tests were performed in the same manner as for baseline sNfL. The Benjamini-Hochberg method was used to correct for multiple comparisons (adjP).

## Results

### Basic characterization of sNfL in BiDirect

**Figure 1:**
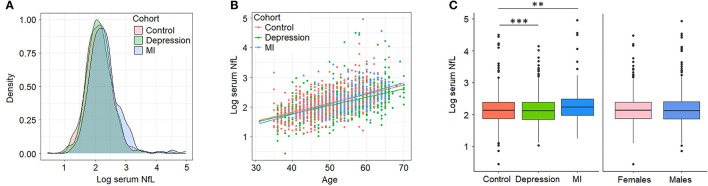
Serum neurofilament light chain (NfL) levels (log2-transformed) in BiDirect. Cohorts showed similar distributions of sNfL concentrations **(A)**. There was a positive correlation of sNfL with age **(B)**. Mean differences associated with the diagnostic group (BiDirect cohort: depression, myocardial infarction-MI-, and population-based control individuals), but not with sex, were observed **(C)**. **p < 0.001, ***p < 0.0001.

Our initial characterization of sNfL in BiDirect found similar distributions of sNfL in the three cohorts (sNfL raw mean ± standard deviation values: control 9.49 ± 6.57, depression 9.24 ± 4.99, MI 11.76 ± 11.62; corresponding log2 values: control 2.15 ± 0.44, depression 2.13 ± 0.43, MI 2.29 ± 0.5; Figure 1A) and a positive association with age (*p* < 2 × 10^−16^, beta = 0.03), which was independent of the cohort (Figure 1B). Age-adjusted comparisons showed mean differences in sNfL levels between both patient cohorts (depression *p* = 8.2 × 10^−5^, MI *p* = 1.4 × 10^−3^) and the reference cohort, while no differences could be attributed to sex (*p* = 0.56) in this dataset (Figure 1C). Moreover, baseline (s0) sNfL correlated well with all other sNfL measurements (i.e., log- and non-transformed values from follow-up visits), and with markers of inflammation, and of the functions of kidneys, liver and thyroid glands (Supplementary material 2, Supplementary Table 1).

### Genetic associations with sNfL

We identified no genetic associations with sNfL surpassing the desired genome-wide significant threshold (*p* < 5 × 10^−8^). But, our observations reached a significance threshold commonly accepted for suggestive associations (*p* < 1 × 10^−5^) in GWASs. Therefore, we wished to further explore these suggestive findings from our GWAS and meta-analysis.

**Figure 2:**
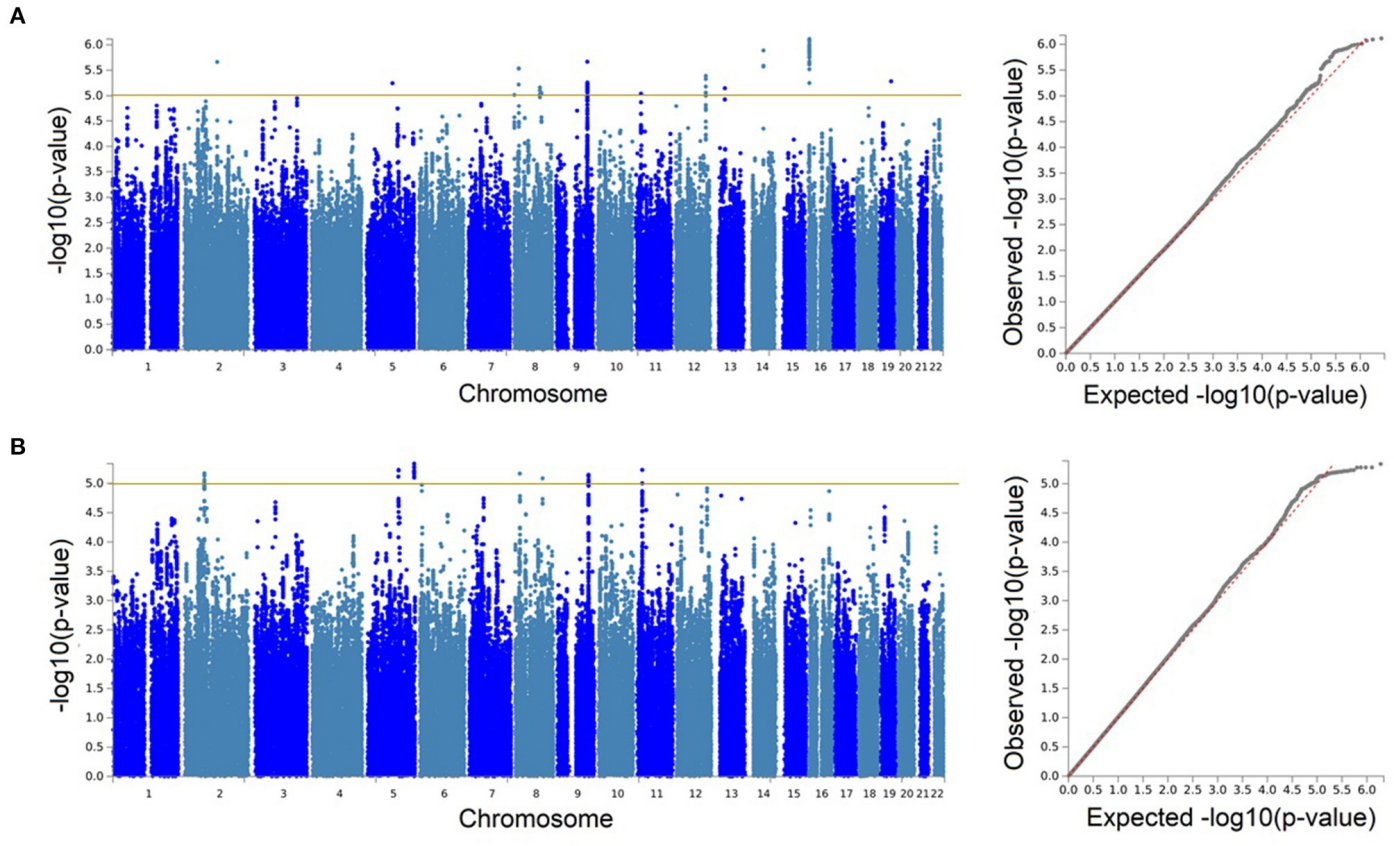
Genetic associations with serum neurofilament light chain were only identified at the suggestive level. Manhattan and quantile-quantile (QQ) plots for the discovery genome-wide association study in BiDirect (*N* = 1,899) **(A)**, and its meta-analysis with ASPS-Fam (*N* = 2,186) **(B)**. Yellow lines in the Manhattan plots mark the suggestive threshold for genome-wide significance (*p* < 1 × 10^−5^).

With our discovery GWAS in BiDirect (*N* = 1,899), we observed suggestive signals in 10 chromosomes (Figure 2A). Because the SNP2GENE tool integrates observations coming from GWAS summary statistics with information on LD structure coming from well-established reference panels to define lead variants and genomic loci, and can also be used to annotate an array of functional features for SNPs within the defined loci, we considered this tool to provide an appropriate means for the interpretation of our results. Twelve suggestive genomic loci for sNfL were defined through this analysis. These loci contained 13 lead variants (i.e., identified from independent variants and independent from each other at *r*^2^ ≥ 0.1), 14 independent signals (i.e., independent variants at the suggestive *p*-value threshold and independent from each other at *r*^2^ ≥ 0.6), and implicated a total of 246 genetic variants and of 18 mapped genes, from which 7 (*CNTNAP5, NAT1, NATP, MTDH, RIMS2, VWA8*, and *RBFOX1*) are protein-coding (Table 2, Supplementary material 2, Supplementary Table 2). The SNP heritability estimation performed with GCTA showed that this GWAS explained about 30% of the variance in sNfL (*h*${}_{SNP}^{2}$= 0.299). However, the analysis also suggested that a larger sample size would be required to confidently detect the genetic component of sNfL (LRT = 2.4, *p* = 0.061).

**Table 2:** Suggestive genomic loci for sNfL measures in BiDirect and the meta-analysis with ASPS-Fam.

**Locus**	**Index variant**	**Alleles**	**Chr**	**Index BP**	**Index P**	**Index effect**	**Start (BP)**	**End (BP)**	**# Variants**	**#Ind.Sig. Variants**	**Ind.Sig. Variants**	**#Lead variants**	**Lead variants**	**Genes (protein-coding)**
**Discovery GWAS (BiDirect;** ***N*** = **1,899)**														
1	rs76037384	T/A	2	125357776	2.17E-06	+	125357776	125403277	23	1	rs76037384	1	rs76037384	CNTNAP5
2	rs12674781	C/T	8	1377915	9.64E-06	+	1356333	1378411	25	1	rs12674781	1	rs12674781	-
3	rs184931198	C/T	8	18210838	2.90E-06	+	17954598	18218371	10	2	rs184931198, rs73198093	1	rs184931198	NAT1, NATP
4	rs142838371	G/A	8	98741426	6.88E-06	+	98656430	98741426	5	1	rs142838371	1	rs142838371	MTDH
5	rs34372929	AT/A	8	104596668	8.87E-06	+	104530581	104718242	7	1	rs34372929	1	rs34372929	RIMS2
6	rs62576696	A/G	9	118311682	2.15E-06	+	118167915	118488131	100	2	rs62576696, rs12380012	2	rs62576696, rs12380012	-
7	rs1842909	C/G	11	18918227	9.10E-06	+	18873142	18939666	25	1	rs1842909	1	rs1842909	-
8	rs146801204	T/C	12	117050196	4.07E-06	–	117039399	117060536	10	1	rs146801204	1	rs146801204	-
9	rs76207901	G/T	13	42524241	7.12E-06	+	42388330	42524241	3	1	rs76207901	1	rs76207901	VWA8
10	rs1514928	C/A	14	62678303	1.29E-06	+	62669677	62678303	3	1	rs1514928	1	rs1514928	-
11	rs8060528	C/T	16	7024428	7.69E-07	–	7011164	7038560	34	1	rs8060528	1	rs8060528	RBFOX1
12	rs74607435	T/C	19	45235700	5.21E-06	+	45235700	45235700	1	1	rs74607435	1	rs74607435	-
**Meta-analysis (BiDirect** + **ASPS-Fam;** ***N*** = **2,186)**														
1	rs34523114	A/AT	2	74131786	6.75E-06	– –	74127289	74140230	42	1	rs34523114	1	rs34523114	ACTG2, TPRKB^a^
2	rs114956339	G/A	5	118578014	5.88E-06	+ +	118365512	118595407	4	1	rs114956339	1	rs114956339	DMXL1
3	rs529938	T/T	5	177961577	4.61E-06	+ +	177959285	177963534	21	1	rs529938	1	rs529938	COL23A1
4	rs73198093	G/C	8	17954598	6.81E-06	+ +	17954598	18107883	8	1	rs73198093	1	rs73198093	NAT1
5	rs34372929	AT/A	8	104596668	8.20E-06	+ +	104530581	104718242	5	1	rs34372929	1	rs34372929	RIMS2
6	rs10982883	T/C	9	118461688	7.14E-06	+ +	118450617	118488131	40	1	rs10982883	1	rs10982883	-
7	rs1842909	G/C	11	18918227	5.89E-06	+ +	18873142	18939666	24	1	rs1842909	1	rs1842909	-

sNfL, serum neurofilament light chain; Chr, chromosome; BP, base pair; Ind.Sig.Variants, individual significant variants.

Loci were defined using FUMA GWAS (LD block *r*^2^ ≥ 0.6, window 500 kb, lead variant *p* < 1e^−5^, clumped variant *p* < 0.05).

^a^eQTL effects of variants in the locus according to the BRAINEAC dataset. ^#^means “number of”.

Because the ASPS-Fam cohort has a small sample size and differences in its composition, in comparison with BiDirect, were evident, we chose not to seek validation of our findings in ASPS-Fam, but to use this cohort to carry out a meta-analysis with the aim to gain statistical power (*N* = 2,186). After performing a weighted meta-analysis and filtering out heterogeneous variants (i.e., variants with inconsistent effects), we applied again the SNP2GENE approach to extract a relevant interpretation of our results. Even with the addition of the ASPS-Fam cohort, we did not observe genomic variants reaching genome-wide significance (Figure 2B). Nevertheless, we were able to define 7 suggestive meta-analysis loci spanning 5 chromosomes, 144 variants and 8 mapped genes, including 6 protein-coding genes (*ACTG2, TPRKB, DMXL1, COL23A1, NAT1*, and *RIMS2*), that associated with sNfL levels in individuals without neurological conditions (Table 2, Supplementary material 2, 3, Supplementary Table 3, Supplementary Figures 1–7). In comparison with our discovery GWAS, meta-analysis loci represented the identification of 4 robust signals (i.e., meta-analysis loci that overlapped GWAS loci; meta-analysis loci #4–7 in chromosomes 8, 9, and 11), as well as the addition of 3 new signals (i.e., meta-analysis loci not found with the discovery GWAS; meta-analysis loci #1–3 in chromosomes 2 and 5). SNP heritability performed with LDSC in our sNfL meta-analysis was estimated to be about 5% (*h*${}_{SNP}^{2}$ = 0.0557). Nevertheless, we observed a low Chi^2^ statistic (mean Chi^2^ = 1.01) for this analysis, which may be due to the small sample size.

### Investigation of biological context

**Figure 3:**
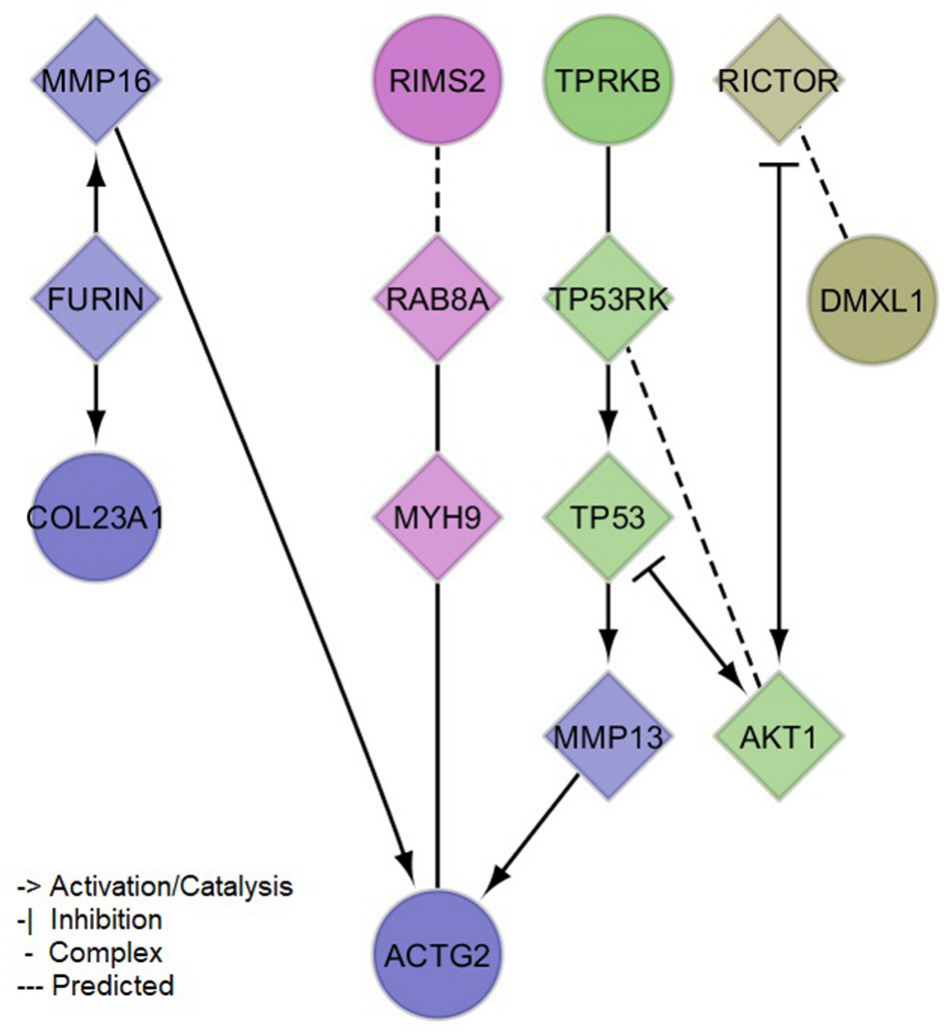
Protein-protein interaction network of mapped and brain expression quantitative trail loci genes implicated by the identified (meta-analysis) suggestive loci for serum neurofilament light chain. Circles denote input genes. Diamonds denote linker proteins. Colors denote network clusters, whose enrichments for pathways and gene ontology cellular compartments can be found in the Supplementary material 2, Supplementary Tables 4, 5.

The PPI network created with the protein-coding genes implicated by our meta-analysis loci was able to link 5/6 (exception of *NAT1*) genes by the incorporation of 9 linker proteins (Figure 3). Four small clusters were defined within this network, which illustrated the differential, yet interconnected functional properties between clusters. The most prominent pathways enriched in each cluster (Supplementary material 2, Supplementary Table 4) were related to cell signaling and organization of the extracellular matrix (lilac module: *ACTG2, COL23A1, FURIN, MMP13, MMP16*), senescence, inflammation and cell death (green module: *AKT1, TP53, TP53RK, TPRKB)*, glucose and insulin metabolism (magenta module: *MYH9, RAB8A, RIMS2*), and immune processes (olive module: *DMXL1, RICTOR*). These pathways showed consistency with the associations observed between sNfL and clinical variables, including not only inflammation but also those related to thyroid and renal functions, and to blood lipids (e.g., Parathyroid hormone synthesis, secretion and action-FDR = 0.0086 in lilac module-; Thyroid hormone signaling pathway-FDR = 0.0052 in green module-; Plasma lipoprotein assembly, remodeling, and clearance-FDR = 0.03 in lilac module). Additionally, network modules were enriched for distinct cellular compartments (Supplementary material 2, Supplementary Table 5), mainly: extracellular matrix and Golgi (lilac module), cytoplasm and nucleus (green module), presynaptic cytoskeleton and transport vesicles (magenta module), and the RAVE (regulator of ATPase of vacuoles and endosomes) and TORC2 (target of rapamycin complex 2) complexes (olive module).

Because none of the variants in our GWAS reached the common threshold accepted for genome-wide significance, we also dissected these associations. For all lead variants from our meta-analysis loci (rs34523114, rs114956339, rs529938, rs73198093, rs34372929, rs10982883 and rs1842909), we found significant differences in sNfL levels from BiDirect participants with different genotypes, particularly in those individuals with two copies of the effect/minor allele (AA genotype), as compared to those homozygous for the non-effect/major allele (BB genotype) (Table 3). With the exception of rs114956339 (*p* = 0.0016), we found no interactions for sNfL measurements between the genotypes of these variants and the diagnostic group (i.e., depression, MI and control).

**Table 3:** Genotype-dependent differences in sNfL for lead meta-analysis variants.

**Locus**	**Variant (rsID)**	**Effect allele (A)**	**Non-effect allele (B)**	**By genotype (P, age adjust)**	**By genotype-group (P, age adjust)**	**AA vs. AB (P, 2–1)**	**AA vs. BB (P, 2–0)**	**AB vs. BB (P, 1–0)**
1	rs34523114	AT	A	7.17E-05	0.596	3.7E-01	2.5E-04	1.4E-03
2	rs114956339	A	G	3.25E-05	0.00157	NA	NA	3.3E-05
3	rs529938	T	C	4.44E-05	0.18	3.6E-04	4.7E-05	6.1E-01
4	rs73198093	C	G	1.06E-05	0.311	NA	NA	1.1E-05
5	rs34372929	A	AT	2.82E-05	0.945	1.5E-03	1.9E-05	1.9E-01
6	rs10982883	C	T	2.78E-05	0.815	1.6E-02	1.2E-04	1.8E-02
7	rs1842909	G	C	9.01E-06	0.594	5.1E-02	8.5E-06	3.8E-03

Summary of ANCOVA results (*p*-values) for comparisons of sNfL values among BiDirect participants according to genotype, and genotype by group interactions. NA, not available.

Finally, we tested the associations of meta-analysis loci with clinical variables. None of these survived correction for multiple comparisons (adj*P* > 0.05), therefore we focused on the top signals (*p* < 0.05) from these tests only. At this threshold, we found evidence suggesting associations of meta-analysis loci with several clinical variables (Supplementary material 2, Supplementary Table 1). When prioritizing these by the integration of our results from genetic association and sNfL correlation tests, we identified overlaps for 18 variables from the clinical phenotypes (Table 4). These included markers of inflammation (interferon-α, and interleukins 6 and 1α), renal function (cystatin, creatinine, albumin and urea), liver and muscle function (lactate dehydrogenase and lipase), thyroid function (free thyroxine and free triiodothyronine), and blood lipids (HDL cholesterol and triglycerides). Noticeably, the index of comorbidity (which included stroke, leg thrombosis, peripheral artery disease, hypertension, MI, diabetes, depression, cancer, kidney and lung diseases, chronic arthritis, and Parkinson's disease) and gray matter volume (relative to total brain, coming from magnetic resonance imaging data) were also prioritized. Moreover, the associations with all sNfL measurements from follow-up visits remained suggested (Supplementary material 2, Supplementary Table 1).

**Table 4:** Prioritized clinical variables in BiDirect showed significant correlations with sNfL and the GWAS meta-analysis loci.

**BiDirect time point**	**Variable label**	**Instrument**	**Effective N**	**# Variants *p* < 0.05**	**sNfL Pearson *p*-value**	**sNfL Pearson coefficient**
dx	Index of comorbidity	Comorbidity	1,899	2	0	0.1889
s0	Gray matter volume relative total brain	(f)MRI	1,208	7	0	−0.2749
s0	HDL cholesterol i.S. mmol/l	Blood lipids	1,849	26	1.40E-03	0.0741
s0	Triglyceride i.S. mmol/l]	Blood lipids	1,850	3	3.60E-02	−0.0488
s0	Interleukin-6 (IL-6) i.S. pg/ml	Inflammation	1,880	7	3.60E-02	−0.0483
s0	Interleukin-1α (IL-1α) i.S. pg/ml	Inflammation	1,880	7	4.10E-02	−0.047
s0	Lactate dehydrogenase i.S. μkatal/l	Liver + muscle function	1,850	38	2.90E-07	0.1189
s0	Lipase i.S. μkatal/l	Liver + muscle function	1,841	21	1.70E-03	0.0732
s0	Cystatin i.S. mg/l	Renal function	1,842	51	0	0.3109
s0	Creatinine i.S. μmol/l	Renal function	1,850	30	0	0.2028
s0	Urea i.S. mmol/l]	Renal function	1,845	31	0	0.197
s0	Albumin in serum (i.S.) g/l	Renal function	1,849	21	3.50E-04	−0.0831
s0	Free triiodothyronine (ft3) i.S. pmol/l	Thyroid function	1,792	1	7.70E-04	−0.0793
s4	Interferon-alpha (IFN-α) i.S. pg/ml	Inflammation	957	21	3.10E-02	−0.0698
s4	Lactate dehydrogenase i.S. μkatal/l	Liver + muscle function	968	26	2.60E-06	0.1504
s4	Creatinine i.S. μmol/l	Renal function	970	4	2.50E-09	0.1899
s4	Urea i.S. mmol/l	Renal function	971	4	1.30E-09	0.1933
s4	Free thyroxin (ft4) i.S. pmol/l	Thyroid function	970	23	8.00E-03	0.0851

^#^means “number of”.

## Discussion

With the increasing interest in the clinical use of sNfL as a peripheral biomarker for the presence, progression and treatment response of neurological conditions in general, there is a need to define which biological factors contribute to physiological variations in sNfL concentrations. Previous studies have reported age, body mass index, blood volume, renal function (as measured by serum creatinine levels), hypertension and pregnancy may act as determinants of sNfL (3–6, 22). To some extent, we corroborated the association of sNfL with aging and renal function, and observed other physiological variables potentially associated with sNfL in the BiDirect study. Nevertheless, because of the small-effect interactions and overlaps at the genetic level that we observed, more studies will be necessary to clarify whether these findings may represent true determinants of serum sNfL levels or an epiphenomenon.

As our primary goal was to determine genetic factors that contribute to modulate sNfL concentrations, we performed a discovery GWAS and meta-analysis study in Europeans. Although we report here the findings from both analyses, we focused on the 7 suggestive loci resulting from our meta-analysis of the BiDirect and ASPS-Fam study populations to gain some biological insights on the implicated genomic regions. Results from our network analysis and overlapping genetic associations with a set of clinical variables show consistency. These highlighted particularly important roles for inflammation, lipids, thyroid hormones and vesicular transport. We also found in the literature, for all protein-coding mapped and/or any-tissue eQTL genes for variants in all of our meta-analysis loci, functions that are relevant for neuronal development and function. As neuronal processes may impact the release of NfL into the CSF and, consequently, its dissemination into peripheral blood, we focused on identifying potential roles of our meta-analysis loci in neuronal functions. However, as suggested by our analyses, it is possible that some variants contribute to regulate sNfL levels through effects on the body's metabolism and renal clearance.

In our study, *NAT1, RIMS2* and *DEC1* (meta-analysis loci #4–6, respectively) were the more robustly suggested candidate genes. The NAT1 (N-Acetyltransferase 1) protein forms an enzymatic complex with ARD1 (N-Alpha-Acetyltransferase 10, NatA Catalytic Subunit; *NAA10* gene) that is required for neuronal differentiation and dendritic arborization (23, 24). The product of *RIMS2* (Regulating Synaptic Membrane Exocytosis 2) functions as a Rab effector involved in synaptic membrane exocytosis (25). *DEC1* (deleted in esophageal cancer 1, *DELEC1*), a lncRNA gene, is a candidate tumor suppressor (26), which means that it may regulate the cell cycle and other fundamental cellular processes.

Moreover, meta-analysis locus #1 mapped to *ACTG2* (Actin Gamma 2) and implicated *TPRKB* (TP53RK Binding Protein) as a brain eQTL gene. Although the ACTG2 protein primarily localizes to the cytoskeleton of enteric smooth muscle, this gene has also been found downregulated during the chemical conversion of cultured human cortical astrocytes into neurons by treatment with small molecules (27), suggesting a role for *ACTG2* in neuronal development. TPRKB is a subunit of the KEOPS (Kinase, Endopeptidase and Other Proteins of small Size) complex, which is required for the threonyl carbamoyl adenosine (t6A) transfer (t)RNA modification (28). An increasing number of reports link defects in these modifications to various neurodevelopmental disorders, suggesting a role in the development of the nervous system (29, 30). Additionally, when looking at any-tissue eQTL effects, genetic variants in meta-analysis locus #1 were found to regulate the expression of *DCTN1* (Dynactin Subunit 1) and *DGUOK* (Deoxyguanosine Kinase). The product of *DCTN1* is essential for the retrograde transport of vesicles and organelles along microtubules mediated by dynein. In neurons, it activates retrograde axonal transport and regulates microtubule stability (31, 32). On the other hand, DGUOK is a mitochondrial protein that may be involved in neuronal differentiation, as suggested by experiments in retinoic acid-induced differentiated neuronal-like cells (33).

Meta-analysis locus #2 mapped to *DMXL1* (Dmx Like 1). In *ngr*1^−/−^ mice, this gene was upregulated in axotomized corticospinal motor neurons 4 weeks after pyramidotomy (34), suggesting a role in axonal repair. Meta-analysis locus #3 mapped to *COL23A1* (Collagen Type XXIII Alpha 1 Chain), whose dysregulated expression has been reported in different brain regions of mice with repeated experience of agonistic interactions (35). The work suggested the involvement of extracellular matrix remodeling (and of *COL23A1*) in the development of experimental psychopathologies. Although meta-analysis locus #7 did not map to protein-coding genes or showed eQTL effects on any in the brain datasets, we found variants in this locus with any-tissue eQTL effects on *PTPN5* (Protein Tyrosine Phosphatase Non-Receptor Type 5). This gene regulates synaptic plasticity, and has been implicated in diverse neurological and psychiatric disorders (36–38).

We acknowledge important limitations of our study. First, the relatively small sample size limited the power to detect genetic associations at the genome-wide level and, therefore, to estimate SNP heritability. This was indeed reflected by the statistics from our heritability analyses. Second, serum samples from non-fasting study participants were used to determine sNfL concentrations. However, it is unknown if fasting status influences sNFL levels. Future assessments of sNfL levels in fasting and non-fasting blood should clarify whether this is a relevant factor for sNfL measurement. And, third, the nature of the study design of the sample populations included in the present study derived in an enrichment of individuals with depression, cardiovascular risk factors and cardiovascular disease. While most prior research focused on neurological conditions, recent studies have shown increased levels of sNfL in patients with cardiovascular or metabolic conditions and multimorbidity (39). In fact, we also showed this to be the case in the BiDirect study. To overcome this, we adjusted for these conditions and other confounding factors, including age. We expect that this is sufficient to adequately address condition-induced biases. Finally, we did not perform analyses within each condition cohort due to their limited sample sizes. Overall, we are positive that the future inclusion of appropriate population-based cohorts will help establish these and other genomic regions as genetic drivers of sNfL variations in individuals without neurological conditions. Further bioinformatics and functional studies should help to elucidate the biological relevance of our findings for sNfL measurements. The potential genetic and physiological factors associated with sNfL that were identified by our study warrant future investigations that will pave the way for an optimal application of sNfL as a marker of neuronal conditions.

## Data availability statement

The data analyzed in this study is subject to the following licenses/restrictions: The summary statistics datasets generated in this study are available from the authors on reasonable request. The derived data supporting the conclusions presented in this article are included within the article and the corresponding additional files. Requests to access these datasets should be directed to MH-R, marisol.herrera@uni-muenster.de.

## Ethics statement

The studies involving human participants were reviewed and approved by the Ethics Committee of the University of Münster and the Westphalian Chamber of Physicians in Münster, North Rhine-Westphalia, Germany and the Ethics Committee of the Medical University of Graz, Austria. The patients/participants provided their written informed consent to participate in this study.

## Author contributions

MH-R: project design, data analysis, interpretation, and manuscript preparation. EH, MK, and RS: GWAS in ASPS-Fam. MS and KB: project design and critical revisions. KB: coordination of the BiDirect study. HW, AM, DL, PB, and JK: measurements of NfL. All authors contributed to the article and approved the submitted version.
