# Supplementary material for: Evidence of polygenic regulation of the physiological presence of neurofilament light chain in human serum
Source: Front Neurol. 2023 Mar 8;14:1145737. doi: 10.3389/fneur.2023.1145737 (PMC10030935; doi:10.3389/fneur.2023.1145737)
Supplement: Supplementary file 1 [file Data_Sheet_1.PDF]

Evidence of polygenic regulation of the physiological presence of neurofilament light chain in human serum

Marisol Herrera-Rivero, PhD, Edith Hofer, PhD, Aleksandra Maceski, MSc, David Leppert, MD, Pascal Benkert, PhD, Jens Kuhle, MD, Reinhold Schmidt, MD, Heinz Wiendl, MD, Monika Stoll, PhD, Klaus Berger, MD.

Supplementary Methods

BiDirect Study

BiDirect is a prospective observational study that integrates three different cohorts: (1) patients with acute depression, (2) patients after an acute cardiac event, and (3) reference subjects from the general population. Starting in 2009 and over the course of 12 years, four personal examinations were conducted. The core examination program, unchanged across follow-ups, comprised a personal interview (e.g. medical diagnoses, health care utilization, lifestyle and risk behavior), a battery of self-administered questionnaires (e.g. depressive symptoms, readiness to change health behavior, perceived health-related quality of life), sensory (e.g. olfaction, pain) and neuropsychological (e.g. memory, executive functions, emotional processing, manual dexterity) assessments, anthropometry, body impedance measurement, a clinical work-up regarding the vascular status (e.g. electrocardiogram, blood pressure, intima media thickness), blood sampling (serum and plasma, DNA), and neuroimaging (e.g. diffusion tensor imaging, resting-state, emotional faces processing). A short

questionnaire covering the perceived health state (and recent changes thereof), recent medical diagnoses, work and living situation, new life events, perceived depressive symptoms, and current medication, is mailed to all participants one year after each of these examinations.

Overall, 2315 participants were recruited for BiDirect-Baseline and 2258 (97.5%) of them matched the final inclusion criteria.

Cohort 1: consisted of 999 patients, who suffered from an episode of depression at the time of recruitment. Recruitment took place at six different psychiatric and psychosomatic hospitals and departments located in and around the city of Münster (radius: 35 km), as well as two resident psychiatrists' practices located in Münster. The recruitment of outpatients was limited to those who had been hospitalized due to depression at least once during the 12 months period prior to inclusion into the study. Inclusion criteria were (i) age (≥35 and < 66 years) and (ii) current in- or outpatient treatment due to acute depression. Exclusion criteria were (i) compulsory admission, (ii) comorbid dementia, and (iii) comorbid drug abuse (including alcohol). Potential participants were ascertained by trained and certified study psychologists, and eligible patients were invited to participate in BiDirect-Baseline. Appointments were scheduled via telephone or email.

Cohort 2: comprised 347 patients with CVD (excluding cerebrovascular disease) who were recruited by trained and certified study nurses in four different cardiology departments and rehabilitation clinics in and around Münster (radius: 50 km). Inclusion criteria were (i) age (≥35 and < 66 years) and (ii) acute myocardial infarction, or acute coronary syndrome requiring therapy, or treatment of cardiac disease due to myocardial infarction within the last three months. In case of consent, the CVD patients

were invited to participate in BiDirect-Baseline two months after recruitment by the study nurse, and appointments were scheduled via telephone or email.

Cohort 3: included 912 community-dwelling adults (age: ≥ 35 and < 66 years). The participants had been randomly sampled from the population register of the city of Münster and were invited for BiDirect-Baseline via letter; appointments were scheduled by telephone or email.

With the exception of the psychiatric interviews, which were administered by trained and certified study psychologists in most cases at the hospitals where recruitment took place, the majority of data was collected by trained and certified study nurses at the BiDirect Study Center, an integral part of the Institute for Epidemiology and Social Medicine at the University of Münster, Germany. The MRI scans were obtained by the staff of the Department for Clinical Radiology at the University of Münster.

The study was approved by the ethics committee of the University of Münster and the Westphalian Chamber of Physicians in Münster, North-Rhine-Westphalia, Germany. Written informed consent for participation in the study was obtained from all participants.

Study protocol describing the rationale and design of the BiDirect Study: Teismann H et al. Establishing the bidirectional relationship between depression and subclinical arteriosclerosis--rationale, design, and characteristics of the BiDirect Study. *BMC Psychiatry.* 14, 174 (2014).

ASPS-Fam

The Austrian stroke prevention family study (ASPS-Fam) is a prospective single-center, community-based study on the cerebral effects of vascular risk factors in the normal elderly population of the city of Graz, Austria [Seiler et al., 2014, Ghadery et

al., 2015]. The ASPS-Fam represents an extension of the Austrian stroke prevention study (ASPS), which was established in 1991 [Schmidt et al., 1994, 1999]. Between 2006 and 2013, study participants of the ASPS and their first-grade relatives were invited to enter ASPS-Fam. Inclusion criteria were no history of previous stroke or dementia and a normal neurologic examination. A total of 419 individuals from 176 families were included into the study. The number of members per family ranged from two to six. The entire cohort underwent an extended diagnostic work-up including clinical history, blood tests, cognitive testing, magnetic resonance imaging and a thorough vascular risk factor assessment. The study protocol was approved by the ethics committee of the Medical University of Graz, Austria, and written informed consent was obtained from all subjects.

For sNfL measurements, all serum samples were analyzed at the University Hospital Basel, Switzerland. Serum NfL levels were determined by single molecule array (Simoa) assay using the capture monoclonal antibody (mAB) 47:3 (initial dilution 0.3 mg/mL; Art. No. 27016) and the biotinylated detector mAB 2:1 (0.1 µg/mL; Art. No. 27018) from UmanDiagnostic transferred onto the Simoa platform.

References:

Seiler, S. et al. Magnetization transfer ratio relates to cognitive impairment in normal elderly. *Front. Aging Neurosci.* 6, 263 (2014).

Khalil, M. et al. Serum neurofilament light levels in normal aging and their association with morphologic brain changes. *Nat. Commun.* 11, 812 (2020).

Hilal, S. et al. Enlarged perivascular spaces and cognition: A meta-analysis of 5 population-based studies. *Neurology.* 91, e832-e842 (2018).
