# Supplementary material for: Evidence of polygenic regulation of the physiological presence of neurofilament light chain in human serum
Source: Front Neurol. 2023 Mar 8;14:1145737. doi: 10.3389/fneur.2023.1145737 (PMC10030935; doi:10.3389/fneur.2023.1145737)
Supplement: Supplementary file 2 [file Data_Sheet_2.PDF]

Evidence of polygenic regulation of the physiological presence of neurofilament light chain in human serum

Marisol Herrera-Rivero, PhD, Edith Hofer, PhD, Aleksandra Maceski, MSc, David Leppert, MD, Pascal Benkert, PhD, Jens Kuhle, MD, Reinhold Schmidt, MD, Heinz Wiendl, MD, Monika Stoll, PhD, Klaus Berger, MD.

Supplementary Figures

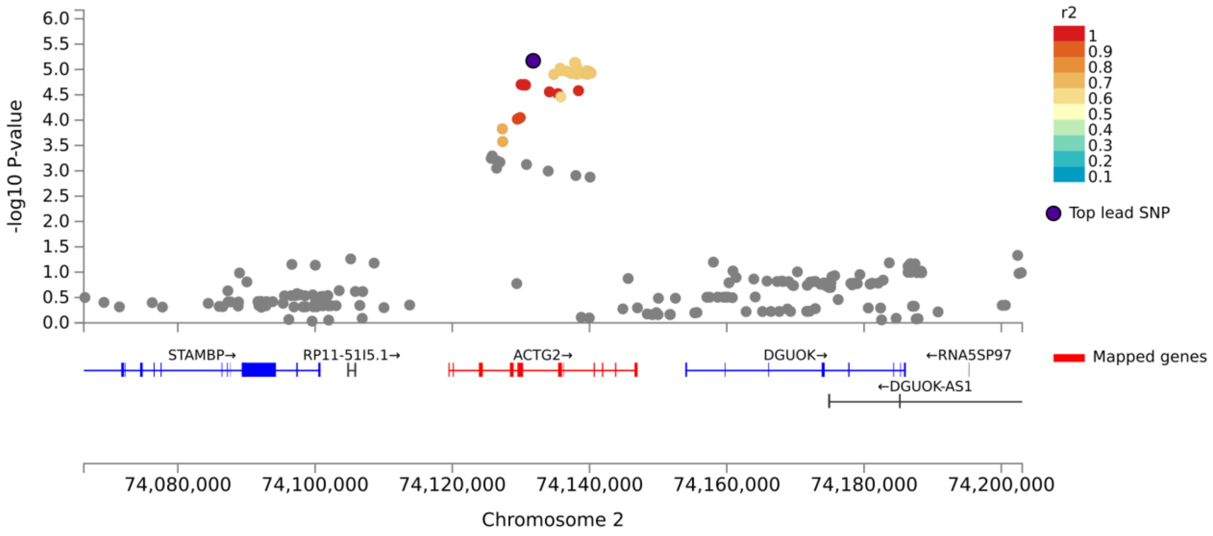

Suppl.Figure.1. Regional association plot for the suggestive locus #1 identified for serum NfL through meta-analysis of the BiDirect and ASPS-Fam studies (N=2,186). Lead variant: rs34523114 ($p = 6.75x10^{-6}$; z = -4.502). Number of supporting variants in locus: 41 ($p<0.05$, $r^2 \geq 0.6$ in 500 kb window from lead variant).

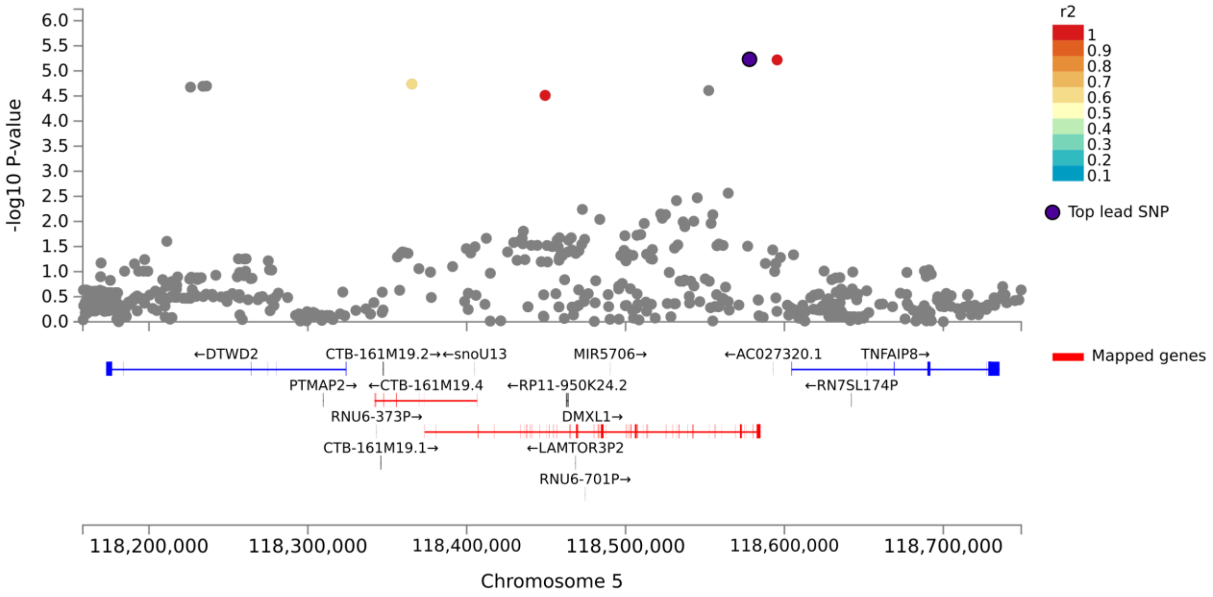

Suppl.Figure.2. Regional association plot for the suggestive locus #2 identified for serum NfL through meta-analysis of the BiDirect and ASPS-Fam studies (N=2,186). Lead variant: rs114956339 (p = 5.88×10^{-6}; z = 4.351). Number of supporting variants in locus: 3 ($p<0.05$, $r^2 \geq 0.6$ in 500 kb window from lead variant).

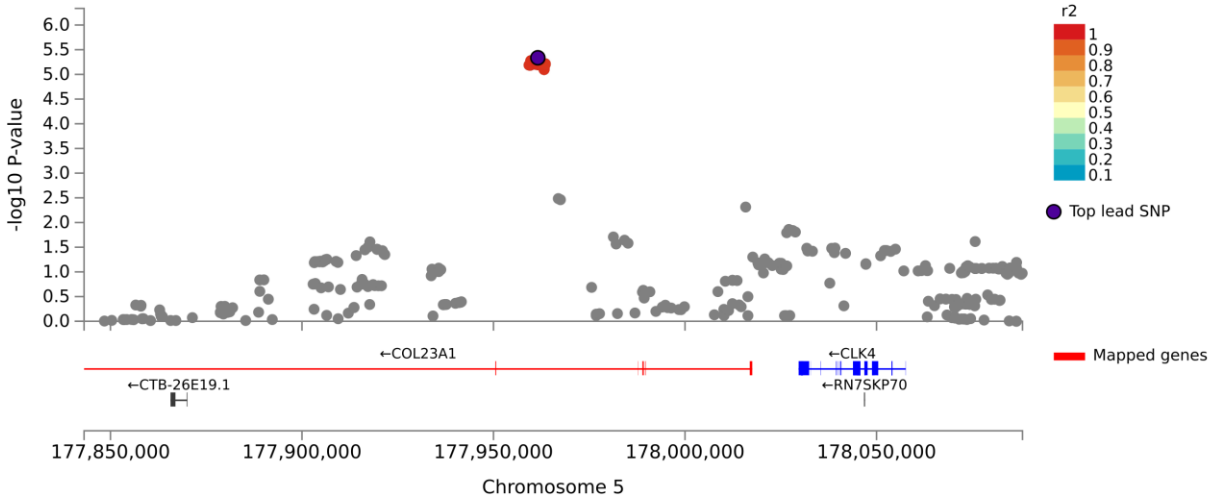

Suppl.Figure.3. Regional association plot for the suggestive locus #3 identified for serum NfL through meta-analysis of the BiDirect and ASPS-Fam studies (N=2,186). Lead variant: rs529938 (p = 4.61×10^{-6}; z = 4.582). Number of supporting variants in locus: 20 ($p<0.05$, $r^2 \geq 0.6$ in 500 kb window from lead variant).

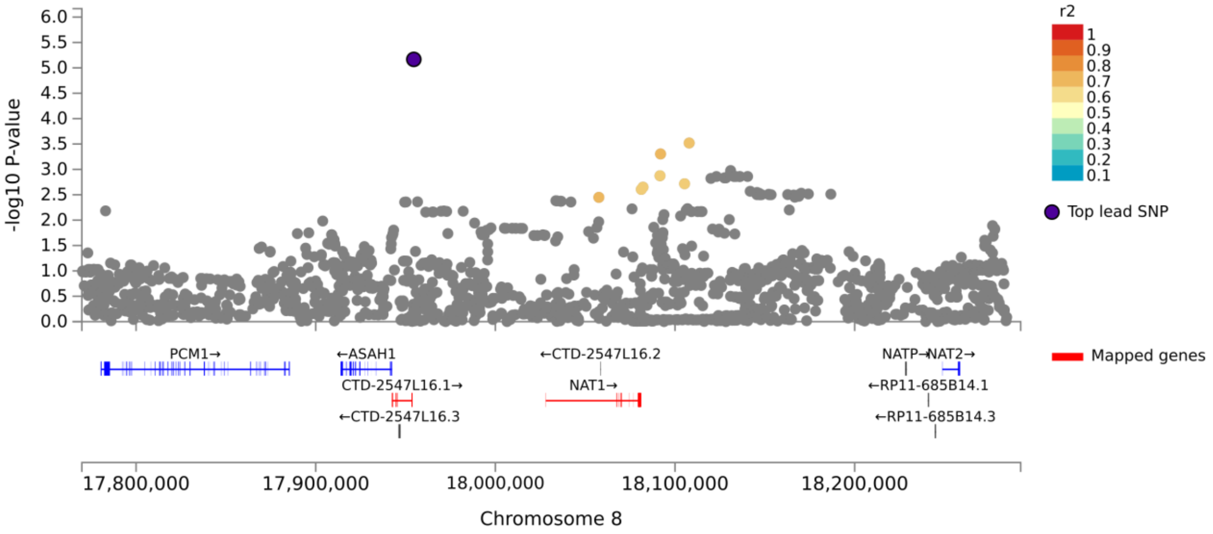

Suppl.Figure.4. Regional association plot for the suggestive locus #4 identified for serum NfL through meta-analysis of the BiDirect and ASPS-Fam studies (N=2,186). Lead variant: rs73198093 (p = 6.81x10^{-6}; z = 4.5). Number of supporting variants in locus: 7 (p<0.05, r^2≥0.6 in 500 kb window from lead variant).

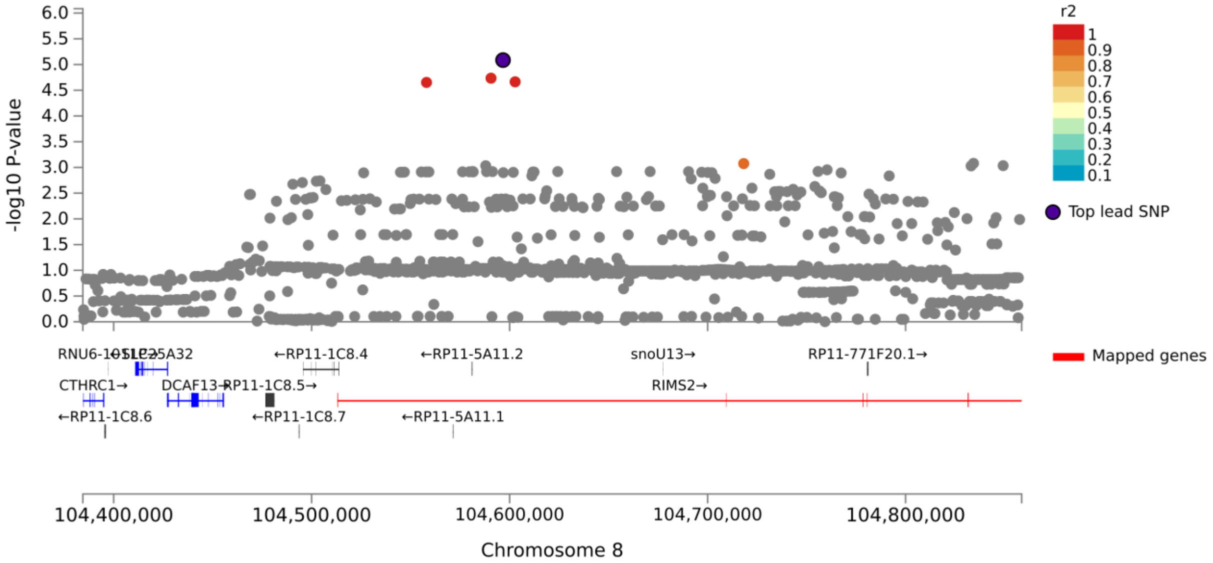

Suppl.Figure.5. Regional association plot for the suggestive locus #5 identified for serum NfL through meta-analysis of the BiDirect and ASPS-Fam studies (N=2,186). Lead variant: rs34372929 (p = 8.2x10^{-6}; z = 4.46). Number of supporting variants in locus: 4 (p<0.05, r^2≥0.6 in 500 kb window from lead variant).

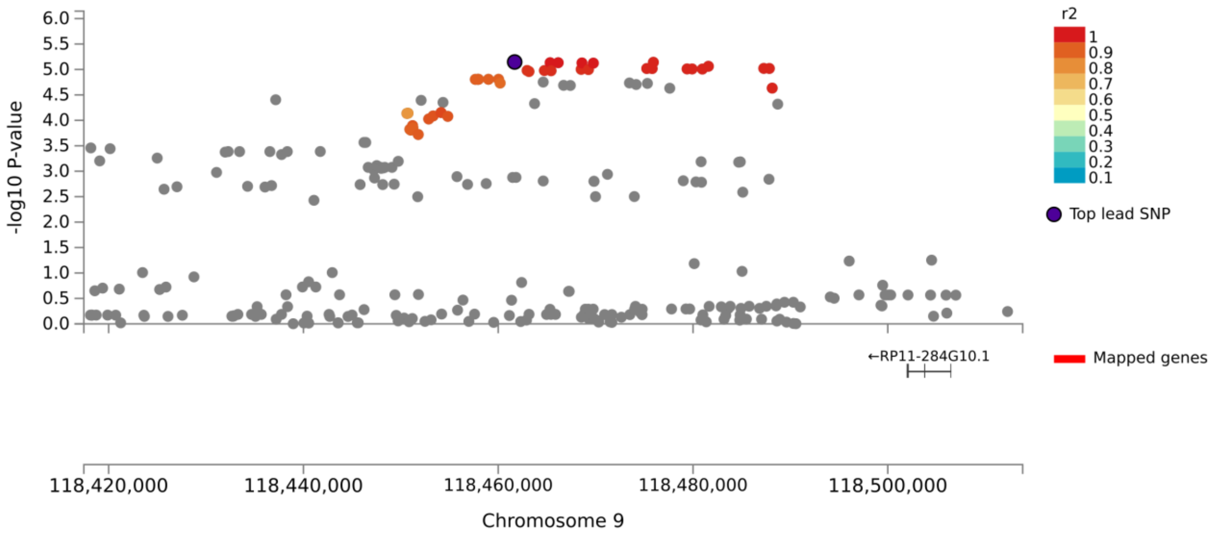

Suppl.Figure.6. Regional association plot for the suggestive locus #6 identified for serum NfL through meta-analysis of the BiDirect and ASPS-Fam studies (N=2,186). Lead variant: rs10982883 ($p = 7.14x10^{-6}$; $z = 4.49$). Number of supporting variants in locus: 39 ($p<0.05$, $r^2 \geq 0.6$ in 500 kb window from lead variant).

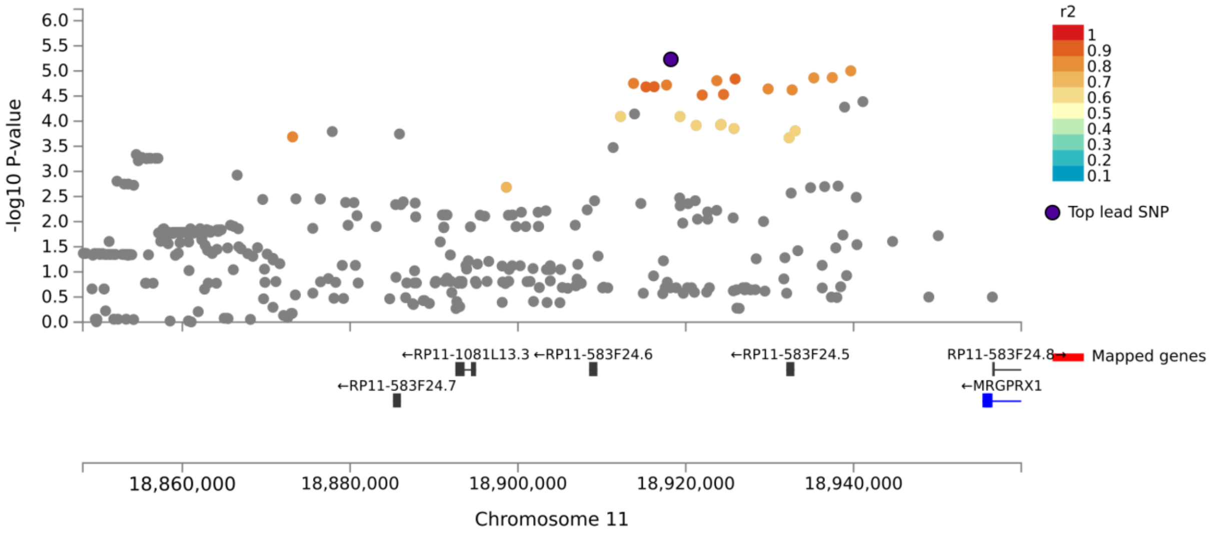

Suppl.Figure.7. Regional association plot for the suggestive locus #7 identified for serum NfL through meta-analysis of the BiDirect and ASPS-Fam studies (N=2,186). Lead variant: rs1842909 ($p = 5.89x10^{-6}$; $z = 4.531$). Number of supporting variants in locus: 23 ($p<0.05$, $r^2 \geq 0.6$ in 500 kb window from lead variant).
